# Repair of Microdamage in Osteonal Cortical Bone Adjacent to Bone Screw

**DOI:** 10.1371/journal.pone.0089343

**Published:** 2014-02-20

**Authors:** Lei Wang, Tingjun Ye, Lianfu Deng, Jin Shao, Jin Qi, Qi Zhou, Li Wei, Shijing Qiu

**Affiliations:** 1 Shanghai Key Laboratory for Prevention and Treatment of Bone and Joint Diseases with Integrated Chinese-Western Medicine, Department of Orthopedics, Shanghai Institute of Traumatology and Orthopedics, Ruijin Hospital, Shanghai Jiaotong University School of Medicine, Shanghai, PR China; 2 Bone and Mineral Research Laboratory, Henry Ford Hospital, Detroit, Michigan, United States of America; Oregon Health & Science University, United States of America

## Abstract

Up to date, little is known about the repair mode of microdamage in osteonal cortical bone resulting from bone screw implantation. In this study, self-tapping titanium cortical bone screws were inserted into the tibial diaphyses of 24 adult male rabbits. The animals were sacrificed at 1 day, 2 weeks, 1 month and 2 months after surgery. Histomorphometric measurement and confocal microscopy were performed on basic fuchsin stained bone sections to examine the morphological characteristics of microdamage, bone resorption activity and spatial relationship between microdamage and bone resorption. Diffuse and linear cracks were coexisted in peri-screw bone. Intracortical bone resorption was significantly increased 2 weeks after screw installation and reach to the maximum at 1 month. There was no significant difference in bone resorption between 1-month and 2-months groups. Microdamage was significantly decreased within 1 month after surgery. Bone resorption was predisposed to occur in the region of <100 µm from the bone-screw interface, where had extensive diffuse damage mixed with linear cracks. Different patterns of resorption cavities appeared in peri-screw bone. These data suggest that 1) the complex microdamage composed of diffuse damage and linear cracks is a strong stimulator for initiating targeted bone remodeling; 2) bone resorption activities taking place on the surfaces of differently oriented Haversian and Volkmann canals work in a team for the repair of extensive microdamage; 3) targeted bone remodeling is a short-term reaction to microdamage and thereby it may not be able to remove all microdamage resulting from bone screw insertion.

## Introduction

Cortical bone screws are commonly used for fracture fixation and bone transplant stabilization. During installation of bone screw, the placement torque inevitably squeezes the surrounding bone, creating microdamage in bone matrix [1], [2], [3]. It has been suggested that microdamage may reduce bone stiffness and strength, thereby increasing bone fragility [4], [5], [6], [7], [8]. Excessive accumulation of microdamage in peri-screw bone is a likely factor contributing to the early loosening of bone screw [9], [10]. Accordingly, the repair of microdamage seems to play a critical role in the maintenance of screw stability.

It is well-known that microdamage is repaired through bone remodeling [11], [12], [13]. Damaged bone matrix is resorbed by osteoclasts, and subsequently the resorption cavity is refilled with new bone formed by osteoblasts [14], [15]. A major function of living bone is to sustain cyclic loading, which causes microdamage accumulation in bone matrix [16]. Meanwhile, living bone can detect microdamage and repair it promptly [14], [16]. Therefore, microdamage is not frequently seen in normal bone. Screw insertion is a mechanical damage that can suddenly generate a large amount of microdamage in surrounding bone [1], [3], [17], thereby resulting in a significant increase in bone remodeling [13], [18], [19]. However, there is little knowledge about the repair mode of microdamage induced by bone screw installation in osteonal cortical bone.

In our recent study, we have found different types of microdamage, including diffuse damage, cross-hatched damage and linear cracks, in canine cortical bone adjacent to bone screw [20]. Diffuse damage, assembled by numerous tiny cracks (<0.5 µm in width), was concentrated at the area neighboring to the bone and screw interface [20]. Larger linear cracks (>10 µm in width), although with much fewer numbers, were coexisted with diffuse or cross-hatched damage or presented independently [20]. There is evidence that initiation of bone remodeling is associated with morphology of microdamage [21]. Larger linear cracks are the primary factor for triggering bone remodeling [21], [22]. In contrast, pure diffuse damage may not accelerate bone remodeling [22]. These results were derived from the studies on fatigue damage of rat cortical bone. It is known that the cortical bone structure is significantly different between rats and larger animals or humans because there is no osteon in rat cortical bone [6]. Likewise, the injury mechanism and morphology of microdamage are also significantly different between bones sustained fatigue loading and screw insertion. We hypothesized that the repair mode of screw-induced microdamage in osteonal cortical bone cannot be deduced from the repair of fatigue damage in rat cortical bone. The objective of this study was to investigate the repair of microdamage in osteonal cortical bone adjacent to bone screw.

## Materials and Methods

Twenty four adult male New Zealand white rabbits (around 3.0 kg) were used in the present study. The animals were randomly divided into 4 groups, 6 in each. The rabbit was anesthetized with pentobarbital sodium (30 mg/kg, i.v.). A 3 cm incision was made at the anterior-lateral aspect of the leg. According to the manufacturer’s instruction, self-tapping titanium cortical bone screws were inserted into the lateral tibial diaphyses. A 1.8 mm hole was drilled perpendicular to the periosteal bone surface, and then a 2.4 mm self-tapping titanium screw was inserted into the prepared hole. After screw installation, the wound was closed by layers. The animals were sacrificed at 1 day, 2 weeks, 1 month and 2 months after surgery. The animal experiment was approved by the Institutional Animal Care and Use Committee (IACUC) of Ruijin Hospital.

A 10 mm long bone segment containing the screw in the middle was cut from each tibial diaphysis. The bone segments obtained from left tibiae were en bloc stained with basic fuchsin, which has been reported elsewhere [23], [24], [25]. Briefly, the bone segments were placed in 1% basic fuchsin in ascending series of ethanol (70, 80, 90 and 100%) for 8 days. After en bloc staining, the bone segments were embedded using methlylmethacrylate (MMA) solutions. After MMA polymerized, two cross sections with 100 µm thickness were cut through the screw hole using an EXAKT cutting/grinding system (EXAKT Medical Instruments, Oklahoma City, OK). The sections were ground down to 50 µm and mounted on the slides.

In basic fuchsin stained sections, microdamage morphology and intracortical bone resorption were examined in the peri-screw bone using a Bioquent image analysis system equipped with fluorescence and bright field microscopes (R&M Biometrics, Inc. Nashville, TN). Peri-screw bone is indicative of the region extending up to 0.5 mm from the bone edge around screw. The measurements included bone edge length (BE.L), bone area (B.Ar), diffuse damage area (DDx.Ar), diffuse damage length (DDx.L) and diffuse damage thickness (DDx.Th) in contact with bone edge, linear crack number (LCr.N), linear crack length (LCr.L), resorption cavity number (RC.N), resorption cavity area (RC.Ar) and distance between resorption cavity and bone edge (RC-BE.Dis). The calculations included fraction of diffuse damage area (Fc.DDx.Ar, = DDx.Ar/B.Ar), fraction of diffuse damage length (Fc.DDx.L, = DDx.L/BE.L), linear crack density (LCr.Dn, = LCr.N/B.Ar), linear crack surface density (LCr.S.Dn, = (LCr.N*LCr.L)/B.Ar), resorption cavity density (RC.Dn, = RC.N/B.Ar) and fraction of resorption cavity area (Fc.RC.Ar, = RC.Ar/B.Ar). The variables for outcome assessments are shown in Table 1.

**Table 1:** The variables for outcome assessment.

Variables	Abbreviation	Unit	Calculation*
Fraction of Diffuse Damage Area	Fc.DDx.Ar	%	DDx.Ar/B.Ar
Fraction of Diffuse Damage Length	Fc.DDx.L	%	DDx.L/BE.L
Diffuse Damage Thickness	DDx.Th	µm	
Linear Crack Density	LCr.Dn	#/mm^2^	LCr.N/B.Ar
Linear Crack Surface Density	LCr.S.Dn	µm^2^/mm^2^	(LCr.N*LCr.L)/B.Ar
Resorption Cavity Density	RC.Dn	#/mm^2^	RC.N/B.Ar
Fraction of Resorption Cavity Area	Fc.RC.Ar	%	RC.Ar/B.Ar
Resorption Cavity Area	RC.Ar	µm^2^	
Distance between Resorption Cavity and Bone Edge	RC-BE.Dis	µm	

*B.Ar: Area of peri-screw bone; DDx.Ar: Area of diffuse damage; DDx.L: Length of diffuse damage; BE.L: Length of bone edge; LCr.N: Number of linear cracks; LCr.L: Length of linear cracks; RC.N: Number of resorption cavities; RC.Ar: Area of resorption cavities.

The basic fuchsin stained sections were also examined using confocal microscopy for qualitative observation of microdamage morphology and bone resorption cavities and the spatial relationship between these two entities.

The differences in mean values among 4 timing groups were compared using one-way ANOVA. Kruskal–Wallis test was used if the variable was not normally distributed. The level of statistical significance was accepted at p<0.05.

## Results

### Microdamage

**Figure 1:**
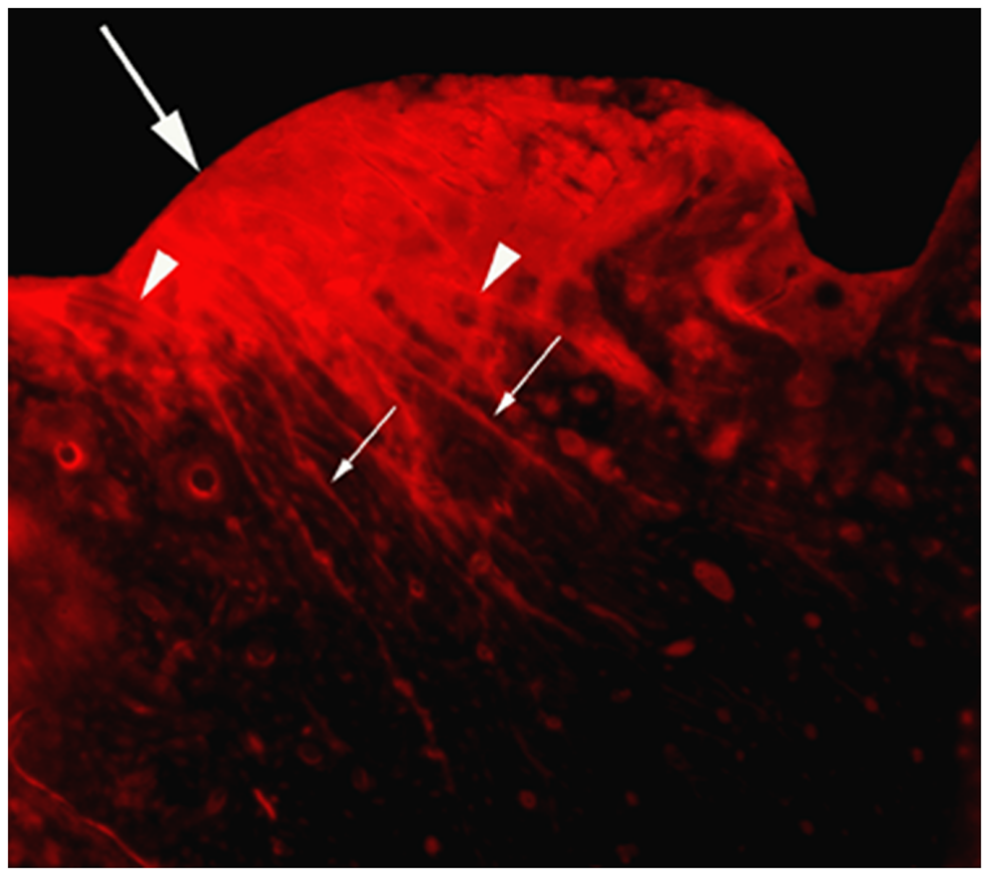
Microdamage in peri-screw bone stained with basic fuchsin. Diffuse damage was contacted with bone edge (large arrow), behind which larger linear cracks (small arrows) were visible. Linear cracks often mixed with diffuse damage (arrow heads) in the area adjacent to the bone-screw interface.

According to the patterns of basic fuchsin stain, microdamage was present as diffuse damage and linear cracks in peri-screw bone. Diffuse damage, including pooled and cross-hatched staining, appeared in a narrow area adjacent to the bone-screw interface. Linear cracks were usually mixed with diffuse damage (Fig. 1), forming a complex microdamage. However, pure linear cracks were often seen in the area behind diffuse damage (Fig. 1).

Both diffuse damage and linear cracks which appeared 1 day after surgery were used as positive control. At 2 weeks after surgery, Fc.DDX.Ar, Fc.DDx.L, LCr.Dn and LCr.S.Dn were evidently reduced, but not reach to statistical significance. These values were all significantly decreased (p<0.05) at 1 month and 2 months after surgery (Table 2). However, there were no significant differences in these variables among 2-weeks, 1-month and 2-months groups (Table 2). In addition, DDx.Th did not show significant difference among 4 groups (Table 2).

**Table 2:** Comparison of microdamage related parameters in peri-screw bone between different time points after surgery.

	Fc.DDx.Ar	Fc.DDx.L	LCr. Den	LCr.S.Den	DDx.Th
1 Day	22.5 (7.61)	67.4 (8.87)	9.96 (3.58)	1621 (529)	125 (26.9)
2 Weeks	14.0 (5.06)	51.0 (12.0)	5.60 (2.67)	824 (461)	100 (26.3)
1 Month	11.5 (5.53)^1^	40.3 (12.2)^1^	3.77 (0.978)^1^	466 (174)^1^	117 (41.8)
2 Months	9.64 (3.99)^1^	36.5 (9.03)^1^	3.09 (1.39)^1^	463 (234)^1^	82.7 (26.3)
P	0.005	<0.001	0.006	0.006	0.144

Data expressed as mean (SD).

Intergroup comparison (p<0.05):

1 Compare with 1 day;

2 Compare with 2 weeks;

3 Compare with 1 month.

### Bone Resorption

**Figure 2:**
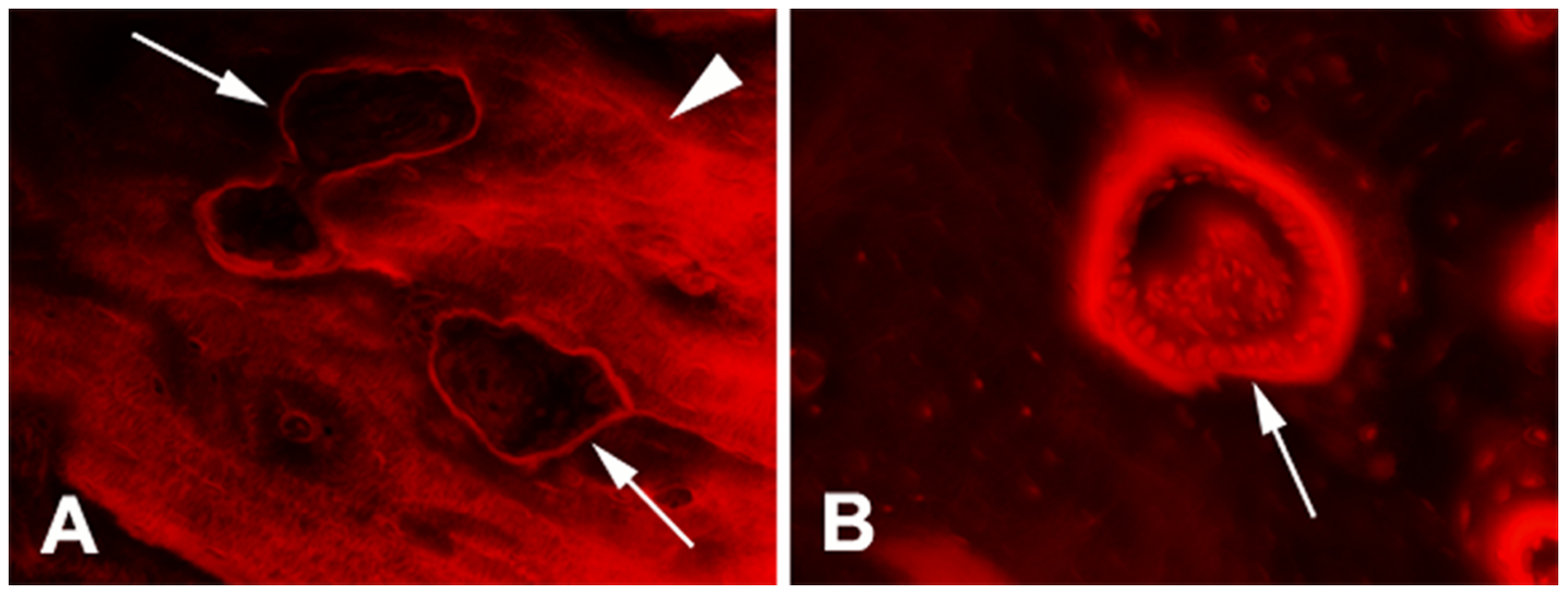
Resorption cavities in peri-screw bone (1 month after surgery). A) cutting cavities (arrow) were covered by a thin layer of basic fuchsin stained tissue and contacted with microdamage; B) closing cavity (arrow) was covered by a thick seem of osteoid, on which there was a single layer of cuboidal osteoblasts attached.

Bone resorption cavities showed two phases during bone remodeling. One was undergoing bone matrix resorption and another was being refilled by new bone. The bone absorbing cavity, termed as cutting cavity, was attached by a thin layer of basic fuchsin stain. The bone refilling cavity, termed as closing cavity, was covered by a thick seam of osteoid, on which a single layer of cuboidal osteoblasts were often visualized (Fig. 2). There were many osteocytes in newly formed bone beneath the osteoid. We referred to both cutting and closing cavities as resorption cavity in this study, because it was often difficult to differentiate these two cavities under the lens of lower magnification used for histomorphometric measurement.

Very few resorption cavities were present in peri-screw bone 1 day after surgery. At 2 weeks after surgery, RC.Dn was remarkably increased and RC.Ar reached to the maximum. RC.Dn and Fc.RC.Ar hit their peak levels 1 month after surgery and declined thereafter. The statistical analyses showed significant differences in RC.Dn (p<0.01), Fc.RC.Ar (p<0.001) and RC.Ar (p<0.01) among 4 groups (Table 3). RC.Dn was significantly increased at 1 month and 2 months compared to 1 day after surgery (all p<0.05). Fc.RC.Ar in 2-weeks, 1-month and 2-months groups was significantly higher than that in 1-day group (all p<0.05). However, Fc.RC.Ar in 2-months group was significantly decreased compared to 1-month group (p<0.05). The peri-screw bone in 2-weeks and 1-month groups contained significantly larger RC.Ar than that in 1-day group (p<0.05). RC-BE.Dis did not show significant difference among 4 groups.

**Table 3:** Comparison of bone resorption related parameters in peri-screw bone between different time points after surgery.

	RC.Dn	Fc.RC.Ar	RC.Ar	RC-BE.Dis
1 Day	0.904 (0.784)	0.361 (0.303)	2452 (2485)	176 (129)
2 Weeks	6.81 (3.31)	7.26 (3.19)^1^	12021 (5431)^1^	153 (43.8)
1 Month	10.6 (3.22)^1^	8.45 (3.34)^1^	8467 (3847)^1^	155 (22.2)
2 Months	8.17 (1.95)^1^	4.10 (1.23)^1^^,^ 3	5085 (1576)	142 (51.2)
p	0.001	<0.001	0.004	0.784

Data expressed as mean (SD).

Intergroup comparison (p<0.05):

1 Compare with 1 day;

2 Compare with 2 weeks;

3 Compare with 1 month.

**Figure 3:**
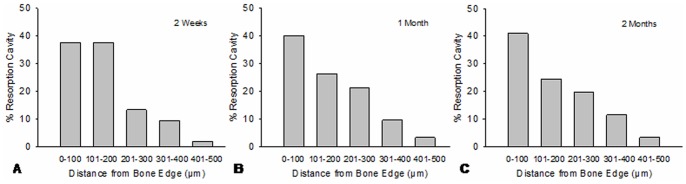
The distribution frequency of bone resorption cavities in different areas of peri-screw bone. The distribution patterns were similar in 2-weeks, 1-month and 2-months groups. In the region of 500 µm from the bone-screw interface, about 40% of the resorption cavities were located in the area of <100 µm from bone edge.

The number of bone resorption cavities decreased with distance from the bone-screw interface. The distribution patterns of bone resorption cavities were similar in peri-screw bone at 2 weeks, 1 month and 2 months after surgery (Fig. 3). In peri-screw bone, about 40% of the resorption cavities were located in a narrow area of <100 µm from the bone-screw interface, which was also the area with diffuse damage accumulation.

### Relationship between Microdamage and Bone Resorption

**Figure 4:**
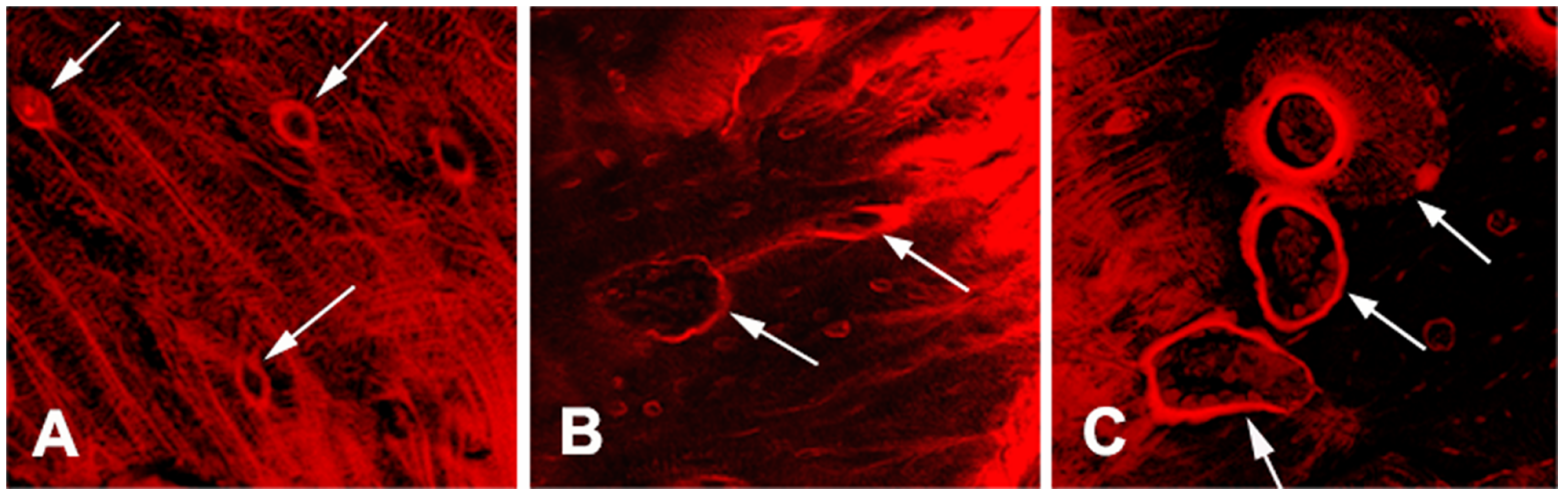
The morphology of microdamage and bone resorption cavities in peri-screw bone. A) microdamage adjacent to the bone-screw interface was composed of diffuse damage and linear cracks (1 day after surgery). The microcracks not only cut through osteocyte lacunae and canaliculi in bone matrix, but also destroyed the surface of Haversian canal (arrows); B) cutting cavities (arrows) in damaged bone (2 weeks after surgery); C) closing cavities (arrows) in damaged bone (2 months after surgery). Extensive osteocyte lacunar-canalicular network was visible in newly formed bone beneath the osteoid (top arrow).

The spatial relationship between microdamage and bone resorption was clearly displayed under confocal microscope. One day after surgery, microdamage was significantly increased in peri-screw bone as compared with the remote area (>2 mm from the bone-screw interface). In bone adjacent to the screw, microdamage mainly consisted of a mixture of diffuse damage and linear cracks, forming a complex microdamage. Such complex microdamage decreased with increasing distance from bone edge. A lot of microcracks invade deep into the osteons and contact with the Haversian canal surface (Fig. 4A). The microcracks also cut through numerous osteocyte lacunae and canaliculi in peri-screw bone.

Resorption cavities were increased in bone with microdamage 2 weeks after screw installation, most of which were cutting cavities and in contact with microcracks (Fig. 4B). Closing cavities were increased in peri-screw bone 1 to 2 months after surgery. Some of the closing cavities were still surrounded by microdamage (Fig. 4C).

**Figure 5:**
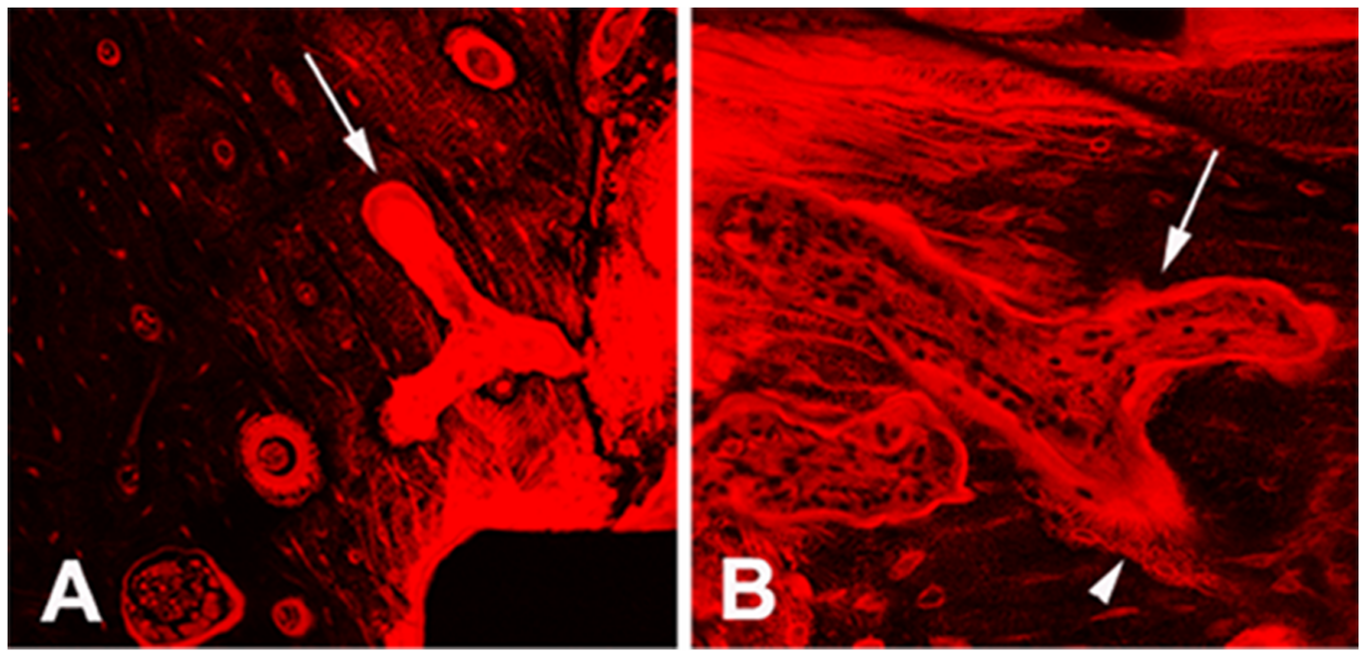
Huge bone resorption cavities in the cross section of peri-screw bone. A) irregular and bifurcated resorption cavity (arrow) in damaged bone; B) Huge resorption cavity (long arrow) with cutting zone (short arrow) in damaged bone and closing zone (arrow head) behind (1 month after surgery).

In the cross section of diaphysis, most of the resorption cavities in bone with microdamage were shaped in circular or oval, but some resorption cavities were huge, irregular and bifurcated (Fig. 5A). The absorbing and refilling phases usually appeared at different locations of the cavity (Fig. 5B).

## Discussion

Although microdamage accumulation and increased bone remodeling are clearly evidenced in peri-screw bone [1], [2], [13], [17], [19], [26], [27], [28], [29], little is known about the repair mode for microdamage induced by screw insertion. Like fatigue damage, both linear cracks and diffuse damage were visible in peri-screw bone [1], [2], [28]. It has been reported that diffuse damage is much less effective for triggering bone resorption than linear cracks, suggesting that the responses to diffuse damage and linear cracks are different [21], [22]. The cracks composed of diffuse damage are at ultrastructural size scope and may not result in severe damage to bone [22]. However, the large-sized linear cracks would cause severe damage to bone matrix, leading to osteocyte apoptosis to initiate bone remodeling [20], [22], [30]. Accordingly, linear cracks are the primary factor contributing to targeted bone remodeling [6], [22].

Yadav et al reported that diffuse damage tended to appear in the area adjacent to the bone-screw interface [1]. It looks confusing that mild diffuse damage appears closely to the bone screw. Our present and previous studies [20] confirmed that diffuse damage in peri-screw bone was frequently mixed with linear cracks, which was referred to as complex microdamage. Such complex microdamage is likely to be caused by the high screw insertion torque that generates overcompression of the surrounding bone [1], [2], [3], [29]. During the process of bone screw installation, large linear cracks are formed quickly to release high energy produced by insertion torque, and then the residual energy adjacent to screw creates tiny cracks to form diffuse damage. Since the energy produced by insertion torque decreases with increasing distance from the bone-screw interface, the density of linear cracks in the area with diffuse damage may be significantly higher than the area without diffuse damage.

Hoshaw et al [19] reported that 4 weeks after installation of dental implant screws many bone resorption cavities were observed in direct association with damaged bone regions. However, they did not notice the relationship between microdamage morphology and distribution of bone resorption in peri-screw bone. In this study, we found that bone resorption became very active within 1 month after screw implantation. Both diffuse damage and linear cracks were significantly decreased in association with increased bone resorption. About 40% of the resorption cavities appeared in bone within 100 µm from the bone-screw interface (1/5 of the peri-screw bone), which was the area concentrated with complex microdamage. Confocal microscopy showed that bone resorption cavities were frequently in contact with microdamage, especially with linear cracks, providing further evidence that linear cracks are the target for bone remodeling [17], [22], [31]. These results suggested that diffuse damage was incidentally removed in the process of repairing linear cracks. Compared to 2 weeks, the size of resorption cavities was significantly reduced 2 months after surgery. The possible reason was that many resorption cavities formed at the early stage were subsequently refilled by new bone [14], [32], [33], leading to a remarkable decrease in the cavity size.

Most of the resorption cavities were shaped in circular or oval in the cross-section of tubular bone, suggesting that they were formed along the longitudinally oriented Haversion canals. Nevertheless, some huge bifurcated resorption cavities were seen in the same section; these cavities seem unlikely originated from the Haversian canals. We assume that such resorption cavities were originated from Volkman canals, which were oriented horizontally and often anastomosed with the Haversian canal [34]. Both Haversian and Volkamann canals serve as the intracortical bone surface upon which bone remodeling is initiated [32], [34], [35], [36]. Unlike Haversion canals, little attention has been paid on the role of Volkmann canals in the repair of microdamage. Microcracks in peri-screw bone were arranged in different orientations [20]. The linear cracks oriented parallel to the longitudinal axis of the osteons may be more easily to be accessed by bone remodeling initiated from the horizontal Volkmann canals than that from the longitudinal Haversion canals. Moreover, Martin [37] proposed that osteocyte apoptosis induced by microdamage may attract or “steer” adjacent existing osteonal resorption spaces to change their destined direction to access damaged bone. This pattern of bone remodeling may participate in the repair of microdamage that is located in the areas remote to the intracortical bone surface, particularly in interstitial bone. Accordingly, microdamage in peri-screw bone is likely to be repaired via a team work of different patterns of bone remodeling.

Based on the results of this study, we suggest that bone remodeling is necessary but not sufficient to remove all microdamage in peri-screw bone. The results showed that the density of bone resorption cavities in peri-screw bone was only increased within 1 month after surgery. There were no significant differences in bone resorption between 1 month and 2 months groups. On the other hand, both diffuse damage and linear cracks were significantly decreased in the regions with increased bone resorption within 1 month after surgery. However, there was no significant decrease in microdamage after 1 month. These results provided strong evidence that targeted bone remodeling is a short-term reaction to microdamage. If microdamage has not been repaired within a relatively short time, it may rarely initiate targeted bone remodeling afterwards. The possible mechanism is related to osteocytes.

There is consensus that osteocytes are essential for the detection and repair of microdamage [11], [21], [38]. Microdamage would rupture the osteocyte lacunae and canaliculi and then damage the cellular material [39], both of which would cause osteocyte apoptosis. The apoptotic osteocytes may send signals to the surrounding osteocytes and cells on the bone surface to initiate bone remodeling [40], [41], [42], [43]. However, apoptosis is a short-term behavior of cells [44]. The release of signals only occurs at the execution phase of apoptotic process [45]. Thereafter, the apoptotic osteocyte will be destroyed and split into multiple apoptotic bodies [43], [46], losing the functions of synthesis and secretion. Many osteocyte lacunae would become empty after osteocyte death [38], [43], [47]. Microdamage in peri-screw bone is extensive and unable to be completely removed by one remodeling cycle. Hence microdamage remained in the region short of osteocytes is unlikely to stimulate targeted bone remodeling for prompt repair. In addition, microdamage located in the region distant from the bone surface, particularly in interstitial bone, is inaccessible by bone remodeling due to its deep-seated position [48]. In these contexts, partial volume of microdamage may remain in bone matrix without being repaired for a long time.

The limitation of this study was to count the complex microdamage as diffuse damage because the mixed linear cracks were often undistinguishable in bone with strongly stained diffuse damage. Therefore, we were unable to differentiate pure diffuse damage from complex microdamage in peri-screw bone, which prevented us from showing the spatial relationship between linear cracks and bone resorption in the area with complex microdamage.

In conclusion, the complex microdamage composed of diffuse damage and linear cracks is located immediately adjacent to the bone-screw interface, which is a strong stimulator for initiating targeted bone remodeling. However, the targeted bone remodeling is a short-term reaction, which is insufficient to remove extensive microdamage in peri-screw bone.
